# New Appliances and Things Medical

**Published:** 1900-06-02

**Authors:** 


### NEW APPLIANCES AND THINGS MEDICAL.

[We shall be glad to receive, at our Office, 28 & 29, Southampton Street, Strand, London, W.C., from the manufacturers, specimens of all new preparations and appliances which may be brought out from time to time.]

### SOAPS AND TOILET PREPARATIONS.

(Edward Cook and Co., Limited, Bow, London, E.)

We have received samples of several different soaps from the above firm. Those with antiseptic and medicinal properties will be of most interest to readers of The Hospital. Cook's gold medal antiseptic soap possesses remarkable germicidal properties, which is naturally to be expected from a soap which contains biniodide of mercury and potassium. The biniodide is skilfully combined with the soap basis in such a way as to be chemically available. For skin diseases which are clearly of microbic origin this soap is very useful, but its application is not limited to therapeutic purposes. As an antiseptic soap for cleaning the hands of surgeons or the skin of patients before operation it is to be highly recommended. Cook's sulphur and glycerine soap is a soothing variety for delicate skins, and in certain forms of eczema and mild psoriasis it can be applied with advantage. The toilet carbolic soap and the compressed coal tar are both preparations which are to be commended where antiseptic properties are sought for in a mild though effective degree. As a really luxurious toilet soap of most delicious perfume there is nothing to beat the Russian Violet, but, though luxurious to a degree, it is perfectly harmless to the most delicate skin, with fine emollient properties. Cook's Riviera soap is a delightfully-scented soap for ordinary toilet purposes; it is particularly pure and economical in use. Their cold cream and oatmeal soap should be used when the water is hard, and is a very successful kind for nursery use.

